# Implementation of Hepatitis Information Management System in Iran

**DOI:** 10.5539/gjhs.v8n6p250

**Published:** 2015-11-10

**Authors:** Safdari Reza, Farzi Jebraeil, Nasiri Ali Akbar, Ghazisaeedi Marjan, Taghavi Mohammad Reza, Afshari Mehdi, Sargolzaee Mahlagha, Taji Farhad

**Affiliations:** 1 Department of Health Information Management, School of Allied Medical Sciences, Tehran University of Medical Sciences, Tehran, Iran; 2 Department of Health Information Technology, School of Allied Medical Sciences, Zabol University of Medical Sciences, Zabol, Iran; 3 School of Medicine, Zabol University of Medical Sciences, Zabol, Iran; 4 Neyshabur University of Medical Sciences, Neyshabur, Iran

**Keywords:** electronic system, hepatitis, health informatics, information management system

## Abstract

**Introduction:**

Nowadays, hepatitis is of the most important health priorities around the world, where information plays a very significant role in specialized diseases prevention planning, and policy- and decision-making processes. Thus, this study addressed challenges of hepatitis information management and investigated the outcomes of establishing a hepatitis information management system to overcome such challenges. To this end, this research intended to study the implementation of an Electronic hepatitis information management system.

**Methodology:**

This is an applied-developmental study with following specifications and procedures: preparation of study proposal and design, justification of the design’s stakeholders, approval of the design by the Postgraduate Education Council of Faculty, determination of pilot hepatitis control center, software development, deciding on control, prevention, and treatment centers, and finally development of a network-based system for collecting and managing hepatitis information.

**Findings:**

Results indicated that the inconsistency and lack of integrity of data, as well as the lack of communication between related units prevented timely information register of viral hepatic patients and services that are provided to them. This inhibited the possibility of considering a follow-up process. However, the implementation of this system and involvement of relevant units greatly solved these problems.

**Conclusion:**

Results show that the implementation of an electronic system for the management of hepatitis control, prevention, and treatment is a regional and national requirement; since, this system with its empowered infrastructure is capable in providing desired services to all laboratories, counseling and health centers, specialized clinics, and physicians connected to the hepatitis network. This enables them to follow up and monitor patients’ conditions. That mentioned system paves the way for the analysis of gathered information, managers’ and specialists’ access in different regions to the data for making appropriate and accurate decisions.

## 1. Introduction

Nowadays, viral hepatitis, especially the blood-borne types (i.e. B and C), and hepatitis prevention are widely recognized significant health priorities, since 5-10% and more than 50% of hepatitis B and C cases develop chronic diseases and liver cancer (Sammak, Azadegan, & Bitarafan, 2010). Hepatitis B is of the main health problems in the world, especially in developing countries. In Iran, it accounts for 70-80% of chronic hepatitis cases and the majority of hepatitis-related mortalities (Asgari & Hagh Azali, 2007). According to the World Health Organization (WHO) and Centers for Disease Control and Prevention (CDC), Iran is among the endemic countries with median prevalence of chronic hepatitis B virus (HBV) infection. In this group of countries, the lifetime risk of HBV infection ranges 20-60% (Poorolajal & Majdzadeh, 2009). Furthermore, another study has reported less than 1% prevalence of hepatitis C virus (HCV) infection (Poorolajal, Mirzaei, Bathaei, & Majzoobi, 2011). Accordingly, HCV prevention in the most affected population, including HIV prone people, is very important as HCV infection facilitates the development of chronic hepatitis, cirrhosis, and liver malignancy (WHO, 2009).

Although the fecal-oral-borne viral hepatitis and water-borne hepatitis (i.e. A and E) do not develop chronic hepatitis, they are responsible for several mortalities and morbidities around the world. Studies in Iran also present their endemic status. Hepatitis A and E are epidemic in countries with low rank on levels of health. Iran is an endemic area for hepatitis A and E infection (Noroozi et al., 2012).

Economically, hepatitis is among the most costly diseases. A study into hepatitis B (acute, intensive, chronic, cirrhosis, and early-stage liver cancer) related outpatients in China, 2010, has estimated a direct treatment cost of 43,311/28#, carrying a significant financial burden on the patient and society (Lu et al., 2013).

An effective disease management requires the disease surveillance system design and implementation to investigate determinant factors in disease incidence and distribution, specify the infected different ages and sex groups and disease process, predict and reach an early diagnosis of epidemics, and contribute to the design and evaluation of health interventions (Safdari, Farzi, Ghazisaeidi, Mirzaee, & Goodini, 2013b; Subhani & Khalid, 2010).

Despite the development of disease management centers in many developed and developing countries, data standardization and creation of registries and healthcare systems structure have been given less attention (Farzi, Salem Safi, Zohoor, & Ebadi Fardazar, 2008). Consequently, an accurate monitoring and evaluation of healthcare process is not possible (NCCMT, 2008 (updated 2013)). In other words, in many countries available information does not suggest the actual prevalence of diseases, as the incidence of the majority of diseases is overestimated (Hummel, 2000).

In this regard, the lack of an information management system for hepatitis control has led to sporadic data collection; as a result, there is not any comprehensive system for gathering, processing, monitoring, and controlling such data (Alavian & Alavian, 2005; Ataei et al., 2011). Accordingly, identification of infected patients, prone groups, geographical distribution, demographic information, and economic, social, human impacts, as well as decision-making and planning for effective interventions are not adequately effective (Lu et al., 2013). Furthermore, data collection process is based on one-way relationships without feedback loops (Asgari & Hagh Azali, 2007). Due to the lack of communication between decision-makers and information providers the efficiency decreases and then confronted with resource loss, duplicate work-low and subjective decisions (Safdari, Farzi, Ghazisaeidi, Mirzaee, & Goodini, 2012, 2013a; Safdari et al., 2013b).

Consequently, this study was conducted aiming at designing and implementing an electronic hepatitis information management system to approach the mentioned limitations.

## 2. Methodology

This is an applied-developmental study with following specifications and procedure: preparation of study proposal and design, justification of the design’s stakeholders, approval of the design by the Postgraduate Education Council of Faculty, adoption of the Control, Prevention, and Treatment Center of Zabol as the pilot center, determination of affiliate centers, analysis of the current and future situation, system design and development, and eventually implementation of a network-based system for collecting and managing hepatitis information.

As a result, after the development of desired software, the measure was taken to implement it in affiliated centers including laboratories, consulting and health centers, and hospitals, under the hepatitis network of Zabol University of Medical Sciences. The aim was to record the information of patients with viral hepatitis from the early diagnosis phase in the system electronically. Indeed, a profile similar to the electronic health record is created, where demographic information, risk factors, infection diagnostic practice, early diagnosis signs, family information, paraclinical measures, signs and symptoms of diseases, physical examination, tests, healthcare plan, and follow-up outcomes are precisely recorded. All users, including nurses and physicians, can record and manage the information if their access to the system is given.

The regional organizations and institutions participating in the research are as follows:

1) The Health Deputy of University
2) The Health Consulting Center
3) The Hepatitis Control, Prevention, and Treatment Center
4) The Blood Transfusion Center
5) The Reference Laboratory
6) The university affiliated health centers

## 3. Findings

After the implementation of the design, following results were achieved: setting up the electronic hepatitis information management system in the Hepatitis Control Center of Zabol and other related health centers at the regional level, connecting the system to referral centers, creation of electronic profile during the diagnosis, treatment, and consulting processes in case of diagnosed infection, providing the affiliates with the system accessibility to get informed about and involved in healthcare team, and preparation of required reports.

After system setting up, one-year information of patients with hepatitis B was recorded from March 21, 2014 to March 21, 2015. Then, the epidemiological information was obtained from the system and analyzed in Excel in form of statistics and tables. A number of this information is provided as some examples.

Table 1 presents one-year data elicited from the system during the mentioned period. It shows the statistical population, comprised of 297 (98.7%) patients with hepatitis B, out of which 179 (60.3%) and 118 (39.7%) subjects were male and female, respectively. Among the 297 HBV infected subjects, 110 patients (37%) were done PCR test, and their disease progress phases were determined. The remaining subjects were not done PCR test and their HBV infection phases were not detected. Results suggested that 7 (6.7%), 6 (5.5%), 88 (79.6%), and 9 (8.2%) out of 110 patients, who were done PCR test, were in the Immune Tolerant HBV Immune Reactive, HBV Interactive Carrier, and HBeAg Negative CHB phases. In addition, their information had been recorded in the system.

**Table 1:** Frequency of HBV based on sex

Diagnosis Sex	Unspecified HBV	HBeAg Negative CHB	HBV Inactive Carrier	Immune Reactive Phase HBV	Immune Tolerant HBV	Total Percentage
Male	112	8	51	6	2	179 (60.3%)
Female	75	1	37	0	5	118 (39.7%)
Total	187	9	88	6	7	297 (100%)

According to the one-year information recorded in the system, the age groups of HBV infected patients were 15-19 to upper than 60. Table 2 depicts that the majority of involved persons are in 20-29 and 30-39 groups with 138 (46.4%) and 80 (27%) patients, respectively.

**Table 2:** Frequency of HBV based on age groups

Diagnosis Sex group	Unspecified HBV	HBeAg Negative CHB	HBV Inactive Carrier	Immune Reactive Phase HBV	Immune Tolerant HBV	Total (Percentage)
0-4	0	0	0	0	0	0 (0%)
5-14	0	0	0	0	0	0 (0%)
15-19	10	0	3	0	1	14 (4.5%)
20-29	95	3	32	4	4	138 (46.4%)
30-39	44	2	30	2	2	80 (27%)
40-49	17	0	12	0	0	29 (9.7%)
50-59	9	2	8	0	0	19 (6.4%)
+60	5	2	1	0	0	8 (2.7%)
Uncertain	7	0	2	0	0	10 (3.3%)

## 4. Discussion and Conclusion

Improvement of health information systems is the first step in progressive health system management capabilities. The healthcare decision makers at every managerial level should gain maximum benefit from information to be capable of making precise data-based decisions and policies, and meeting managerial requirements of healthcare service providers (Kuo & Fuh, 2011; Safdari et al., 2012, 2013a, 2013b).

Results showed that the majority of developed and developing countries require such systems for promoting the level of health, making correct policies and developing micro and macro plans. They have managed to meet their needs by setting up information management and record systems. The electronic system for hepatitis information management, like any other system software such as operation systems and database management systems, should be periodically improved to either eliminate its errors or add new functions (Galindo, 2006). To be explain more, both rapid increase in the volume of transactions in the healthcare system (Safdari et al., 2013b), and supporting new systems with unimproved hardware and software reduced the performance to an unacceptable level (Avison & Fitzgerald, 2006). Therefore, an infrastructural improvement may be needed to expand the capacity or fix performance-related problems (Kusek & Rist, 2004). Paying attention to the opinions of information system staff and users, which may refer to a need for performance improvement, is essential for the completion of the system’s developmental process (Dennis, Wixom, & Roth, 2006). Computers and networks function very complicatedly and technically; thus, the performance and set up problems require an exact scrutiny to determine the best solution for the problems.

EpiTrax software is an example for performance challenges in system implementation; since, contrary to its rapid and successful implementation, it has not become fully operational, due to system performance problems, especially when large forms are attached to the cases. The other reported challenge is connected with data accessibility and the applications of business intelligence system (Simon & Powers, 2004).

The electronic healthcare system for specific diseases is also associated with performance limitations including short history of the company development, limited management capabilities such as patient tracking and reporting (Safdari et al., 2013b), and uncertain performance in centralized patient record systems (Simon & Powers, 2004).

It is noticeable that the evaluation process in form of system epidemiological output analysis is among the dynamic and [currently] developing methods (Brender, 2006). Regarding that information technologies are constantly and rapidly developing and that hepatitis control, monitoring, and treatment centers are naturally data-oriented or technological with variety of activities, results of this study will be modified, and will required the improvement of healthcare systems, technologically.

Considering that Sistan and Baluchestan Province, along with Golestan Province, has the highest rate of hepatitis B (38/3) (Alavian, 2015 April), the electronic hepatitis information management system was set up in Hepatitis Control Center of Zabol. To assess the output capability of this information, data of 297 patients with HBV infection, going to the center for healthcare visit, was fed to the system and its output was analyzed for evaluation. The analysis of results showed that 110 (37%) out of 297 HBV patients were done PCR test and their disease progress phase was detected. The remaining 167 (67%) patients was not determined their infection phase. This can be attributed to long duration of final diagnostic process, costly procedure of the tests, diagnostic measures and economic problems (Lu et al., 2013), limited access to resources (Wallace, McNally, & Richmond, 2008), unawareness about the intensity of the problem (Asgari & HaghAzali, 2007), and general incapability of available information system in monitoring, evaluation, and follow-up processes (Farzi et al., 2008).

In this study, 60.3% of the infected people were male, and the majority of the involved patients were in the 20-29 age group. Other studies also suggest infection age reduction in recent years (Alavi-Naini, Sanei-Moghadam, Khosravi, & Salahshour, 2011; Momen-Heravi & Akbari, 2011). This may refer to increased rates of hepatitis B risk factors in lower age groups in recent years.

On the one hand the main research objective is the design and implementation of healthcare information management system for patients with viral hepatitis in regional Hepatitis Prevention, Control, and Treatment Centers. On the other hand, addressing this and similar subjects is a mission of healthcare system at different healthcare and policy levels. So, it is concluded that national micro and macro policy-making and planning in the healthcare area urgently require integrated, comprehensive, accurate, and updated information. This is not possible without the necessary information systems.

Due to the lack of information integrity, information inconsistency, and disconnection between relevant units and service provision authority, viral hepatic patients’ information is not recorded and controlled timely. Although, by operating this system and collaborative participation some of the problems are solved, involvement of specialists and researchers in the areas that hepatitis information is recorded and management is required. In general, findings of this study suggest the hepatitis information management system as a solution to many challenges and problems of patients, physicians, and decision-makers in healthcare area. For further improvement and development, this study provides following recommendations: broader participation of relevant specialists, and greater coordination among healthcare policy- and decision-makers in implementation of specified and approved tasks. These allow us to take more effective step in prevention, control, and treatment of viral hepatitis and to perform more efficiently in favor of national health promotion.

Additionally, cooperation among laboratories, the blood transfusion centers, and academic network affiliated consulting, healthcare, and medical centers in accurate, timely, and software-based information record and delivery can greatly contribute to the reduction of time and money expenditures.

Another important point is helping patients through covering all medical and particularly specialized laboratory measures by responsible organizations via trust infrastructure. Equipping the Healthcare System with an electronic system for specialized service providing can help it in supporting the patients registered in the designed information management system by providing accurate information of them. Finally, the use of this system not only prevents significant financial burden on the society but also facilitates and accelerates the follow-up and healthcare processes.

## References

[ref1] Alavi-Naini R, Sanei-Moghadam E, Khosravi S, Salahshour H (2011). Changes in risk factors of HBsAg positive blood donors in Zahedan, Iran. Zahedan Journal of Research in Medical Sciences.

[ref2] Alavian S. M (2015). Hepatitis B is a Serious Health Problem in Some Parts of Iran; Sistan and Baluchestan Province. Int J Infect.

[ref3] Alavian S. M, Alavian S. H (2005). Hepatitis D Virus Infection;Iran, Middle East and Central Asia. Hepat Mon.

[ref4] Asgari F, HaghAzali M (2007). National Guideline for Hepatitis B care.

[ref5] Ataei B, Babak A, Yaran M, Kassaian N, Nokhodian Z, Meshkati M, Adibi P (2011). Hepatitis c in intravenous drug users: seroprevalence and risk factors. Journal of Isfahan Medical School.

[ref6] Avison D, Fitzgerald G (2006). Methodologies for developing information systems: A historical perspective The Past and Future of Information Systems:1976–2006 and Beyond.

[ref7] Brender J (2006). Handbook of evaluation methods for health informatics.

[ref8] Dennis A, Wixom B, Roth R (2006). Systems Analysis and Design.

[ref9] Farzi J, Salem Safi P, Zohoor A. R, Ebadi Fardazar F (2008). The study of national diabetes registry system: model suggestion for Iran. Journal of Ardabil University of Medical Sciences & Health Services.

[ref10] Galindo J (2006). Fuzzy databases: Modeling, design and implementation: IGI Global. http://dx.doi.org/10.4018/978-1-59140-324-1.

[ref11] Hummel J (2000). Building a computerized disease registry for chronic illness management of diabetes. Clinical Diabetes.

[ref12] Kuo K.-L, Fuh C.-S (2011). A Rule-Based Clinical Decision Model to Support Interpretation of Multiple Data in Health Examinations. Journal of medical systems.

[ref13] Kusek J. Z, Rist R. C (2004). Ten Steps to a ResultsBased Monitoring and Evaluation System: A Handbook for Development Practitioners: World Bank-free PDF.

[ref14] Lu J, Xu A, Wang J, Zhang L, Song L, Li R, Lu M (2013). Direct economic burden of hepatitis B virus related diseases: evidence from Shandong, China. BMC health services research.

[ref15] Momen-Heravi M, Akbari H (2011). Persistance of HBsAg and serum activities of liver enzymes among chronic carriers of hepatitis B. Zahedan Journal of Research in Medical Sciences.

[ref16] NCCMT (2008(updated 2013)). Planning and assessment tool for chronic disease prevention and management.

[ref17] Noroozi M, Moradi F, Hasanzadeh A, Banazadegan R, Basiri M. R, Ramezani A, Hamkar R (2012). Seroprevalence of Hepatitis A and Hepatitis E in Qom Province, 2011. Iranian Journal of Infectious Diseases.

[ref18] poorolajal j, majdzadeh r (2009). Prevalence of Chronic Hepatitis B Infection in Iran. Iranian Journal of Epidemiology.

[ref19] Poorolajal J, Mirzaei M, Bathaei S. J, Majzoobi M. M (2011). Hepatitis B and C Infections in Hamadan Province during 2004-2009. Journal of Research in Health Sciences.

[ref20] Safdari R, Farzi J, Ghazisaeidi M, Mirzaee M, Goodini A (2012). Intelligence risk detection models: tools to promote patients safety level. Paper presented at the in Proceedings of the 3th Symposium for E-Hospital and Telemedicine.

[ref21] Safdari R, Farzi J, Ghazisaeidi M, Mirzaee M, Goodini A (2013a). The Application of Use Case Modeling in Designing Medical Imaging. Information Systems.

[ref22] Safdari R, Farzi J, Ghazisaeidi M, Mirzaee M, Goodini A (2013b). Healthcare Intelligence risk detection systems. Open Journal of Preventive Medicine.

[ref23] Sammak H, Azadegan H, Bitarafan M (2010). Prevalence of Hepatitis B, C and HIV in Patients with Major ßThalassaemia in Qom, 2007. J Qom Univer Med Scien.

[ref24] Simon J, Powers M (2004). Chronic Disease Registries: A Product Review.

[ref25] Subhani S, Khalid A.-R (2010). Design and development of a web-based Saudi National Diabetes Registry. Journal of diabetes science and technology.

[ref26] Wallace J, McNally S, Richmond J (2008). National hepatitis B needs assessment.

[ref27] WHO (2009). The growing threats of hepatitis B and C in the Eastern Mediterranean region: A call for action. Technical Paper EM/RC56.

